# Decoding the digital: a corpus-based study of simplifications and other translation universals in translated texts

**DOI:** 10.3389/fpsyg.2025.1517107

**Published:** 2025-05-16

**Authors:** Muhammad Afzaal, Baorong Huang, Dina Abdel Salam El-Dakhs

**Affiliations:** ^1^Institute of Language Sciences, Shanghai International Studies University, Shanghai, China; ^2^Institute of Language Sciences, Prince Sultan University: Language and Communication Research Lab, Prince Sultan University, Riyadh, Saudi Arabia; ^3^School of Foreign Languages, Huaihua University, Huaihua City, Hunan, China

**Keywords:** translation universals, visualization tools, corpus-based study, computing, education

## Abstract

Translation universals have garnered significant interest across disciplines such as translation studies, linguistics, and machine translations. However, their exploration remains relatively underrepresented within the realm of technology and computing. Leveraging statistical analyses, principal component assessment, and advanced visualization tools, this study delves into translation universals found within a novel corpus of articles, originally from English journals and magazines, translated by Chinese computing experts. This exploration discerns overarching patterns, termed translation universals, along with nuances distinctive to the computing sector. Furthermore, the research offers theoretical insights into the impetuses guiding such translations, anchored by the entropy theory, the Principle of Least Effort, and the Principle of Relevance. Notably, while the translated versions manifest a higher type-token ratio than their native Chinese counterparts, characteristics of simplification and explicitation remain salient in the translated Chinese text.

## Introduction

In corpus linguistics research, language features and their comparisons are prevalent. The significance of language features, spanning from lexicography to grammar in analyzing discourses, registers, and varieties, has been astutely articulated by Biber et al. (1998). In her study of the Translational English Corpus (TEC), Baker (1995) proposed several features to measure the deviations of translational English text from its original English version. These include metrics like STTR (standardized type-token ratio), mean sentence length, ratio of transitions, and reporting structures. Language features are vast and ever evolving. Over time, linguists have concentrated on myriad new variables in the corpus-based studies of Chinese translational texts, along with the verification of translation universals. These include n-gram analysis, connectives analysis, and investigations into passive or specific constructions (Xiao and Hu, 2015). Further explorations encompass reporting structures, direct speech/free indirect speech (Huang, 2015; Jia et al., 2022), the BEI construction (Hu, 2016), and specific verbs such as “jinxing” (Dai, 2016). To address the challenge posed by the burgeoning number of features, various analytical methods have been proposed. These include multivariate analysis, cluster analysis (Moisl, 2015), and triangulation (Baker and Egbert, 2016).

According to Delaere et al. (2012, p. 326), researchers delving into translation universals have struggled to conclusively demonstrate that observed distinctions between non-translated and translated texts are indeed attributed to the posited translation universal, rather than other underlying factors. This observation remains even though disparities between non-translated and translated texts have been consistently noted. In this academic milieu, Halverson (2003) introduced the Gravitational Pull Hypothesis, aiming to elucidate specific nuances related to the traits of human cognition. Halverson deduced that numerous patterns, initially perceived as exclusive to translation, are more plausibly natural by-products of bilingual language production rather than intrinsic features of the translation process (Halverson, 2013, p. 50). Echoing a similar sentiment, House (2008, p. 11) posits that translation universals are untenable, arguing that any behavior discerned in the translation process inherently extends to all linguistic activities.

Against this backdrop, this study focuses on the translation universals and hypotheses of gravitational pull hypotheses to elucidate the prevailing trends and distinctive features in technical Chinese translations. Furthermore, it advocates for a provisional amalgamation of information theory from communication studies, the Principle of Least Effort, and the principle of relevance from pragmatics. This integrated approach aims to probe the intentional choices professionals make regarding wording and syntactic patterns that exhibit normalization, simplification, and explication in the translation of technical Chinese within the computing domain.

### Literature review

Earlier studies have explored various aspects of translation (e.g., Huang, 2007; Malmkjær, 2011; Mu et al., 2015; Tukey, 1977; Wang and Qin, 2009; Wang and Hu, 2008; Wein, 2023; Alotaibi, 2020; ELAffendi and Alrajhi, 2022; Shah et al., 2024). Of particular relevance to the current study is the extensive examination of the translated texts which led to the formulation of the explicitation hypothesis. In the 1970s, Toury (1980, p. 60) proposed that translated texts tend to elucidate information implicit in the source text “irrespective of the translator’s identity,” suggesting that the specific identity of the translator is inconsequential. Blum-Kulka (1986) explored this propensity and discovered that translated texts contained more cohesion markers than their non-translated counterparts. Baker (1996, p. 180) posited that translated texts often “spell things out rather than leave them implicit,” with this tendency being particularly prominent in lexis and syntax. Supporting evidence indicated that translated texts frequently elucidate ambiguities instead of leaving interpretations to readers. The notion of translation universals, as proposed by scholars like Toury (2004), Baker et al. (1993), Kruger (2002), and Lin et al. (2023), has been profoundly explored in the context of Chinese-English translation. While translation universals have been studied across various domains, including politics (Hu et al., 2015), translation (Wang et al., 2023), literature (Hu, 2015), and business (Feng et al., 2018), technical Chinese translation in China remains unexplored.

Laviosa uses the term “convergence” to describe “leveling off,” defining it as the “relative homogeneity of translated texts in terms of their scores on given measures of universal features.” This stands in contrast to non-translated texts, which manifest greater idiosyncrasies and variance (Laviosa, 2002, pp. 72–73).

Chesterman (2004) categorized these features into two distinct groups: S-universals and T-universals. The “S-universal” pertains to generalizations derived from comparing source texts with their translations, encompassing patterns such as explicitation, normalization, and standardization. In contrast, the “T-universal” alludes to generalizations drawn from juxtaposing translations with non-translations in the target language, with simplification serving as a prime example. Regarding syntactic complexity, Liu and Afzaal (2021) delved into translation universals, examining the simplification hypotheses within corpus-based translation research. Their inquiry hinged on two disparate yet analogous corpora: the English monolingual segment of COCE (Corpus of Chinese-English) and the indigenous English corpus of FLOB. Both are accessible online (Freiburg-LOB Corpus of British English) (Afzaal and Du, 2023; Afzaal et al., 2022; Liu and Afzaal, 2022).

Normalization embodies the tendency among translators to align with patterns and practices characteristic of the target language, sometimes even accentuating them. This proclivity is termed “normalization” (Baker, 1996, p. 176). Baker contends that the use of “typical grammatical structures, punctuation, and collocational patterns or clichés” serves as markers of normalization (Baker, 1996, p. 183; Baroni and Bernardini, 2006). Notably, contemporary research on this attribute has employed diverse markers. Recent investigations largely bifurcate into two segments: those focusing on phrasal-level markers and others concentrating on syntactic-level indicators.

The phenomenon of translation universals (TUs) has been investigated by several studies. However, although recent studies validate TUs across various language pairings and registers, some questions remain unanswered (Tirkkonen-Condit, 2002; Afzaal, 2022; Jia et al., 2022; Wang, 2023). In addition, several studies fail to clearly validate the overarching theories of translation universals, particularly regarding terminology. For instance, Toury (2004) and Chesterman (2004) argue that the most definitive assertion about translation universals is their conditional and probabilistic manifestation. Halverson (2003) emphasizes that the cognitive foundations of translation provide a broader framework for explanation and generalization, suggesting that translation universals constitute a secondary tier of generalization. Nevertheless, these conditional assertions about translation universals can be instrumental in identifying overarching patterns (Chesterman, 2004, p. 43).

On the other hand, a language universal is an attribute purported to be consistent across all languages. Given that around 6,000–7,000 languages are spoken globally, any identified language universals must be both highly general and abstract. Often, these universals are formulated using mathematical constructs, as exemplified by Croft et al. (1990). Yet, hypotheses regarding language universals can be tested against a plethora of global languages. In contrast, the notion of translation “universals” presents a different landscape. The entirety of translations ever produced—past and present—is immensely varied. Consequently, claims about “universals” in translation research demand more nuanced interpretations. Given this complexity, some scholars prefer alternative terms like general trends, patterns, or even generalizations, as long as they are suitably qualified and conditioned.

Tymoczko (1998), along with other researchers, pointed out a related concern. When establishing a corpus of translations to formulate or test hypotheses about universals, there is a need to clearly define what qualifies as a translation. Questions arise: Should we include translations by individuals fluent in the target language, or should we limit the scope to works by certified experts? What about translations by amateurs, fan-made translations, or those done by children? Furthermore, the challenge extends to deciding on the inclusion criteria for adaptations, variations, and other derivative works.

The crux of this criticism suggests that we ought to forgo making sweeping universal claims that cannot be substantiated. Instead, we should focus on discerning the circumstances in which certain generalizations, albeit not universally applicable, might hold true. This implies moving away from overarching assertions that lack empirical support. For instance, while some universal claims have been debunked in their broadest sense, they may still hold as conditioned generalizations within particular translation types or modes. Another point of contention is that theories concerning universals have, thus far, been tested on only a limited set of language pairs or combinations.

This paper seeks to address the following research questions:

1. Are there translation universals present in the works of professional computing translators?
2. What drives these translation universals?
3. What underlies the discrepancies observed in translation universals?

### Methodology

This study employed the CCF (China Computer Federation) corpus to investigate whether translational universals encompassed normalization, explicitation, and simplification. It analyzed technical Chinese translations crafted by professionals, using reference materials provided by the same group of experts. This approach aimed to uncover both the overarching trends in Chinese translations and the unique characteristics of individual articles. To achieve this, the research utilized general variables from corpus linguistics, such as STTR (standardized type-token ratio), mean sentence length, the lexical-to-function word ratio and lexical richness. Additionally, specific variables were incorporated to shed light on syntactic complexity. These included the pronoun ratio—a notable metric in English-Chinese corpus translation studies—the ratio of transitions, syntactic richness, and the adposition ratio.

Visualization tools played a crucial role in our analysis. Pairwise scatterplots were employed initially to delve into the attributes of individual translations, highlighting the interplay between pairs of variables. Furthermore, we utilized PCA—a tool for dimension reduction—and its associated visualizations to provide a clear comparison between original Chinese text and its translated counterparts.

This study also presented findings based on the corpus, striving to elucidate the motivations behind the universals using the information theory, the Principle of Least Effort, and the Principle of Relevance in pragmatics. A thorough explanation of the corpus’s unique features and the specific analytical methods applied in this study is provided in the subsequent sections.

### Corpus of the study

The corpus data comprises articles sourced from the Communications of CCF over a span of 5.5 years, from January 2017 to June 2022 (inclusive). The Communications of CCF (CCCF) offers insights into advancements in computer science to members of the China Computer Federation (CCF). Its primary audience includes engineers and researchers in the field of computing. Professionals in computing contribute the articles featured in these magazines, and a specialized translation committee oversees the editing process. Each edition of CCCF includes a translation column showcasing papers from prestigious publications, such as Science and Communications of the ACM. These papers are translated by professors and PhD candidates in computing, and their names, along with their research interests, are cited in the translated pieces. The quality of translations in CCCF is further reinforced by a dedicated editorial committee comprising four professionals, led by a senior CCF member who also holds the title of IEEE Senior Member.

The corpus data is bifurcated into two sections. The reference segment contains original Chinese articles from CCCF contributed by computing professionals. In contrast, the translation text includes the Chinese translations of articles provided by the same group of professionals featured in the magazine. Given that both the Chinese and translated articles come from the same cadre of esteemed professionals—rather than typical translators—the corpus forms a robust foundation for contrasting the stylistic nuances between original and translated technical Chinese texts.

For this corpus, only technical Chinese articles in unique columns, distinguished topics, and viewpoints within CCCF are chosen to ensure precise comparisons with their translations. A standard article in CCCF comprises a title, summary/abstract, several keywords, a body, footnotes, and references. However, for the sake of simplicity, this corpus includes only the main content of the article. Elements such as section titles, footnotes, citation numbers, figures, and tables within the body have been omitted. After this preprocessing, 725 articles were shortlisted, with 588 being original Chinese pieces and 137 being Chinese translations.

### Annotations procedure

In this paper, we utilized Stanza, a “language-agnostic fully neural pipeline for text analysis, which covers tokenization, multiword token expansion, lemmatization, part of speech and morphological feature tagging, dependency parsing, and named entity recognition”. It supports multiple languages, including English, German, Arabic, Russian, and Chinese. Descriptive language studies rely on statistics collected through various tools developed by researchers in natural language processing (Afzaal et al., 2024; Lei et al., 2024).

Our annotation pipeline comprised sentence segmentation, tokenization, and POS tagging. We considered general variables in corpus linguistics, such as STTR, mean sentence length, and the lexical-to-function word ratio. For instance, when discussing the use of pronouns in translated Chinese text, Xiao and Hu (2015, p. 101–103) noted that pronouns tend to appear in the translated Chinese text due to the influence of existing pronouns in the source English text. Thus, we included the ratio of pronouns as an indicator in this paper, along with the ratio of transitions and the ratio of adpositions (prepositions and postpositions). These indicators highlight the syntactic simplifications that professional translators might employ.

Furthermore, to measure the information load in a sentence, we proposed to use entropy, a concept introduced by Shannon (1948). According to Shannon, entropy is the function of the possible variables, as represented in Equation 1:


(1) $$H=-K\sum_{i=1}^{n}p_{i}\log p_{i}$$

where K is a positive constant. This equation implies that the information load in a message is determined by the potential choices of its components. In other words, the information load in a sentence is shaped by the possible syntactic and paradigmatic choices of its elements, with the best choice being statistically determined based on the premise that “anyone speaking a language possesses, implicitly, vast knowledge of the statistics of that language” (Shannon, 1951).

## Results and discussions

In this study, we calculated the entropy of each article based on Scipy (Virtanen et al., 2020), a python package for scientific computation. Table 1 displays the general variables in both the Original Chinese Text (OCT) and the Translated Chinese text (TCT) for comparative analysis. OCT comprises 588 articles, containing 1,941,210 tokens across 59,643 sentences. Within this corpus, 22,234 pronouns, 22,775 transitions, and 104,083 adpositions are identified. In contrast, TCT consists of 137 articles, 346,560 tokens, and 13,053 sentences, with 9,945 pronouns, 6,686 transitions, and 19,739 adpositions. Together, the corpora total 725 articles, 2,287,770 tokens, and 72,696 sentences, with 32,179 pronouns, 40,286 conjunctions, and 123,822 adpositions.

**Table 1:** General corpus statistics.

Type	OCT	TCT	Total
No. of articles	588	137	725
No. of tokens	1,941,210	346,560	2,287,770
Sentences (total)	59,643	13,053	72,696
No. of pronouns	22,234	9,945	32,179
No. of transitions	22,775	4,686	27,461
No. of adpositions	104,083	19,739	123,822

Table 2 presents a comprehensive comparison of linguistic metrics between OCT and TCT conditions, revealing significant differences across multiple dimensions, using several Mann–Whitney U tests. OCT consistently shows lower standardized type-token ratios per 1,000 words (0.38 vs. 0.41), and higher mean sentence lengths in characters (54.42 vs. 41.81) compared to TCT, all with large effect sizes (Cohen’s d ranging from-1.354 to 1.372). Additionally, OCT exhibits higher lexical-to-function word ratios (2.27 vs. 1.89) and lower pronoun ratios (1.2% vs. 3.2%), indicating more content-focused language and fewer pronouns, respectively. The differences in the ratio of transitions (1.15% vs. 1.32%) and the ratio of adpositions (5.3% vs. 5.7%) are also statistically significant but with smaller effect sizes (Cohen’s d ranging from-0.36 to 0.43). These findings underscore distinct stylistic and structural differences between OCT and TCT texts, providing insights into how language varies across different conditions or datasets.

**Table 2:** Linguistic metrics across multiple dimensions.

Type	OCT	TCT	U	*p*	Effect size (Cohen’s d)
Standardized type-token ratio (1,000 words)	0.38 (0.03)	0.41 (0.03)	22,787	**	−0.75
Mean sentence length (in characters)	54.42 (11.32)	41.81 (5.61)	69,186	**	1.11
Lexical-to-function word ratio	2.27 (0.29)	1.89 (0.21)	69,358	**	1.37
Pronoun ratio	1.2%(0.008)	3.2%(0.015)	7,275	**	−2.4
Ratio of adpositions	5.3%(0.010)	5.7%(0.009)	31,996	**	−0.36
Ratio of transitions	1.15% (0.004)	1.32% (0.004)	45,073.5	**	−0.43
Lexical richness	5.563 (0.189)	5.552%(0.178)	42,347	0.35	0.06
Syntactic richness	2.157 (0.038)	2.187 (0.029)	21,615	**	0.81
Entropy	8.12 (0.27)	8.07 (0.26)	45,569	*	0.30

*indicates *p* < 0.05. **indicates *p* < 0.01.

In the Table 2, lexical richness shows no significant difference, but the syntactic richness shows considerable difference, which also means that the translators use more diverse syntactic structures to render the translated text. The central idea is that although the translators make considerable efforts in simplification, but the text is not simple than that of original Chinese, because of the difficulty in technical translation. The general corpus variables discussed above highlight broad trends in the Chinese translational text compared to the original Chinese text. These trends evidence notable differences in STTR, mean sentence length, pronoun ratio, and the ratio of transitions. However, focusing solely on mean values or general trends might mask variations in individual cases. Outliers can distort the mean value, either elevating or reducing it. This concern has been recognized within the corpus linguistics community, but it requires thorough exploration and appropriate handling. Questions arise, such as determining which factors most influence human perception of a text as a translation, or whether all translations in the corpus deviate in consistent ways from the original text. These questions remain open for researchers to address.

**Figure 1:**
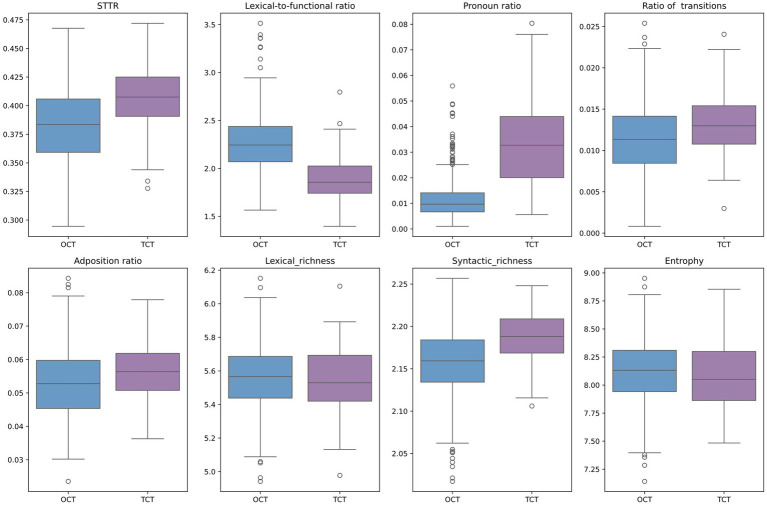
Comparison of the variables in TCT and OCT.

As illustrated in the boxplots in Figure 1, distinguishing between individual original and translated articles linearly is challenging. Assuming the existence of a non-linear plane in the high-dimensional space that encompasses features like STTR, mean sentence length, lexical-to-function word ratio, pronoun ratio, adposition ratio, ratio of transitions, lexical richness, syntactic richness and entropy, this paper utilized principal component analysis to reduce these dimensions to 2D. This reduction aimed to ascertain whether the styles of OCT and TCT are genuinely distinct.

According to Dunteman (1989, p. 7), principal component analysis helps to “both simplify and impose some structure on the research domain… by reducing the number of variables from *p* to a much smaller set of k derived variables. This reduced set retains most of the information of the original *p* variables.” This is achieved by “linearly transforming an original set of variables into a substantially smaller set of uncorrelated variables that represent most of the information from the original set.”

For this study, the authors chose to reduce the six original dimensions in the corpus analysis to two dimensions. This 2D visualization offers an intuitive understanding of the statistical distribution of the variables for individual articles and provides insights into the stylistic differences between OCT and TCT.

**Figure 2:**
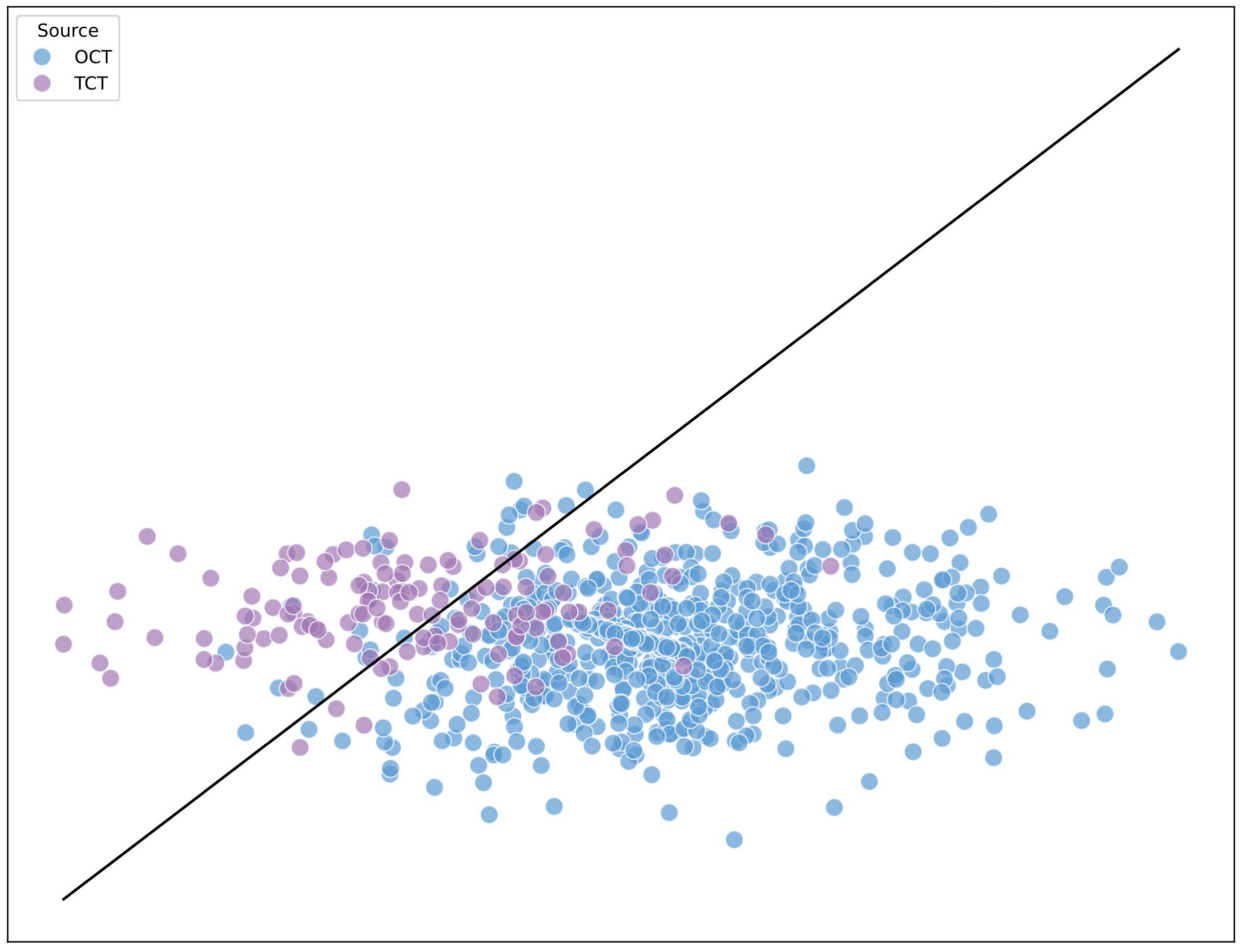
PCA analysis of the variables in OCT and TCT.

For the principal component analysis, we used PCA from scikit-learn (Pedregosa et al., 2011), an open-source Python machine learning library that provides an array of machine learning algorithms. In addition, scikit-learn is used for data mining, and supports supervised and unsupervised learning such as classification, regression, and clustering (Yating et al., 2025). The results were visualized using Matplotlib, another open-source Python library, as depicted in Figure 2. In addition, Matplotlib also offers animated visualizations, high-quality line plots, bar charts and customizations such as labels, legends, colors and styles (Barbara et al., 2024).

The visualization in Figure 1 suggests that there might not be a distinct boundary separating the translated Chinese text (in purple) from the original Chinese text (in blue). Even though the translated Chinese articles tend to cluster near the bottom-left corner and the distribution of the original Chinese text is more diverse, a significant portion of the translated articles falls within the range of the original Chinese articles. This pattern might suggest that professional translators in computing have largely achieved normalization of the translated text.

### Case studies

#### Simplification and explicitation

When translating technical English texts, simplification and explicitation are employed to alleviate the information load for readers encountering unfamiliar materials. The difference of 10.62 characters in the mean sentence length evidences simplification which is different from the study of Jones (2019) on literary translation and translations. This suggests that professional translators often break up a single original English sentence into multiple Chinese sentences in their translations.

1. *Source: Table* *1* *summarizes recent U.S. motor vehicle death rates, which translate to a fatality rate of 5 × 10-7/h per vehicle, assuming an overall average speed of 40 MPH.* (Lala et al., 2020)

*Translation: 表1总结了美国近期的机动车事故死亡率。假设车辆的平均速度为每小时40英里, 则每辆车的致死概率为5 × 10–7/小时。*(He and Ge, 2020).

*Similar Text in OCT: 为了进一步对比不同的控制方式，表1中列举了集中式控制、分散式控制与分布式控制的各项性能指标。从表1可以看出, 在灵活性、可扩展性、可靠性等方面, 分布式控制要远比集中式控制与分散式控制性能更优。 (English translation: In order to further compare different control methods, Table* *1* *lists the performance indexes of centralized control, decentralized control and distributed control. As can be seen from Table* *1**, in terms of flexibility, scalability and reliability, distributed control is far superior to centralized control and decentralized control)*.

In the above example, the translation of one source sentence contains two target sentences that are separated by “。,” the period in Chinese, which contributes to the shorter sentence length in the statistical results. In addition, “assuming (假设)…” is brought to the start position in the second sentence in the translation, which conforms to the ordinary syntactic pattern in China. As shown in a similar text in OCT, the sentence is longer than that of the translated Chinese sentence, which packs more information in a single sentence.

Explicitation is reflected in the lower lexical-to-functional ratio (1.87) in TCT, which means that the translators explicitly showed syntactic clues with more functional words.

1. *Source: Self-certification of the Boeing 737 MAX led to the MCAS system, at the center of the two crashes, being declared non-safety-critical.* (Lala et al., 2020)

*Translation: 例如，波音737 MAX的机动特性增强系统就采用了“自认证”方式，后来引发了两起空难。事实证明，“自认证”难以保证系统安全。* (He and Ge, 2020).

*Similar Text in OCT: 我们经常以为自己的判断和决策都是来自自己的意识，而大量的事实证明并非如此:在判断和决策过程中，通常无意识是主角，意识只是配角，但意识却误认为自己是主角。(English Translation: We often think that our judgment and decision-making come from our own consciousness, but a large number of facts prove that this is not the case: in the process of judgment and decision-making, the unconscious is usually the leading role, while the consciousness is only a supporting role, but the consciousness mistakenly thinks that it is the leading role)*.

In the above example, aside from the sentence splitting, the translators added “例如 (for example, VERB), 就(ADV), 了(PARTICLE), 后来 (later, NOUN)” that established sign posts for readers to untangle the logical and temporal relations among these clauses, thus explicitly revealing the relations buried in the original English sentence. In a similar original Chinese text, the same phrase *事实证明 (proof by facts)* is used, but the sentence is much longer with much larger information load.

Explicitation is also evidenced by the higher pronoun ratio, higher conjunction ratio and higher adposition in the translated Chinese text thus explicitly revealing the syntactic relations in the translated text.

1. *Source: As shown in the accompanying figure, we identify nine potential biases.* (Silva and Kenney, 2019)

*Translation: 如图1所示，我们识别了9个潜在的偏见。*(Jiang et al., 2020).

*Similar Text in OCT: 如图1所示，从该文章出发，向右衍生出若干关键文献。(English Translation: As shown in Figure* *1**, starting from this article, some key documents are derived to the right)*.

In the above example, the translation achieved two types of explicitation. First, it concretized “the accompanying figure (附图)” to “图1 (Figure 1),” an explicit sign post for readers to easily retrieve that figure in the article. Secondly, it uses a subject “我们,” which is transferred from the original subject “we.” In a similar original Chinese text, the original sentence contains no pronoun and no passive voice which is completely grammatical in Chinese.

However, the explicitation of pronouns, transitions, and adpositions in the translation contradicts the hypothesis of normalization to some extent. A normalized translation should have a pronoun ratio similar to that of OCT. Moreover, the higher STTR in TCT seem to contradict the simplification hypothesis, as a simplified version would typically result in a lower STTR in translation. The motivations underlying these contradictions will be explored in the following section.

#### Normalization and explicitation

During the translation process, translators, as bilingual speakers, implicitly process knowledge of the statistics of both the source and target languages. When making lexical, semantic, and syntactic choices, they typically encounter information with heightened uncertainty. In such instances, they might produce translated text with unique elements that diverge from general patterns or norms in the target context, surprising the readers and increasing the entropy in the text with unforeseen items. On the other hand, they can normalize the text, removing all inconsistencies and thus enhancing readability. Both approaches, referred to as literal translation and liberal translation, are entirely valid and are often employed in translation.

In essence, the entropy of the text produced by translators is higher than that of the original text in the target language. It is the translators’ choices that can either increase or decrease the processing efforts required by target readers. Suppose that a translator encounters a sentence like “we incorporate users because their actions affect outcomes” (Silva and Kenney, 2019), a machine translation produced by Google is “我们纳入用户是因为他们的行为会影响结果,” which is comprehensible. However, in Chinese, reasons often precede results, and it is illogical for 我们 (we), the human beings, to incorporate another group of human beings. Thus, in translating this simple sentence, the end results “因为 (because) 用户的行为会影响结果，我们在模型中包含了用户 (Jiang et al., 2020)” not only normalize the original English sentence, but also explicitly add “模型 (model)” to show the relationship between the entities in the sentence.

In other scenarios, translators may choose a more radical strategy to follow the normal pattern, or statistical tendencies, in the target language. For example, the sentence below also contains the same pronoun “we”:

1. Source: What Can We Learn from the Aviation Example With Respect to Autonomous Vehicle Dependability Requirements? (Lala et al., 2020)

Translation: 自动驾驶汽车如何从航空案例中借鉴系统可靠性经验? (He and Ge, 2020)

The translation summarized the original English sentence by dropping “we” and changing “Requirements (需求)” to “experience (经验)” because the collocation of “learn (借鉴)” with “Requirements (需求),” if not forbidden, sounds awkward and foreign to the common Chinese people. In this translation, the translators performed an ingenious maneuver that lowers the lexical surprises, thus reducing the processing efforts for the readers.

In other words, the translators for CCCF, a magazine in technical communication that aims to popularize concepts, viewpoints, and trends in computing, work diligently to introduce new ideas in ways that sound natural to the Chinese audience. They achieve this by simplifying the syntactic complexities of the English language, reducing comprehension efforts through strategies like normalization and explicitation, and adhering to norms in Chinese technical translation that emphasize both accuracy and fluency (Liang, 1996; Li, 1986). They also employ recommended skills such as deletion and summarization (Yan, 1996). The results of such deletion and summarization contribute to higher STTR ratio. The introduction of new information and items from the external world suggests a low chance of repetition, and the processes of deletion and summarization further decrease this repetition. However, as shown in Table 2, the small effect size (0.30) in entropy between the translated Chinese text and the original Chinese text may suggest that, despite the simplification and explicitation used by professional translators, the information load required for readers to understand the translated Chinese text remains on par with that of the original Chinese text. This phenomenon warrants further analysis and explanation.

#### Balanced least effort, relevance and contradictions in translation universals

In an earlier study, Zipf (1949) established the Principle of Least Effort to investigate behavior of translators. This principle suggests that individuals strive to minimize total work when solving problems. Later on, Sperber and Wilson (1986) introduced the communicative principle of relevance, asserting that the information sent by communicators should avoid any disturbances that might lead to varied interpretations.

In translating articles for CCCF, translators play the role of the speaker in Zipf’s speaker-auditor model. Their economy is maximized if they adopt a word-for-word translation approach, as it demands minimal effort to adjust word orders to align with the norms of the target language. Conversely, readers, acting as auditors in Zipf’s model, achieve maximum economy if the translation lacks any exotic elements that deviate significantly from conventional language patterns.

Given that the translators of the CCCF corpus are professionals in this particular domain, it is reasonable to assume that they have the proficiency to translate in the same way they would produce original Chinese texts. For instance, in the translation of “The Internationale,” the pronoun from the original text is omitted in the Chinese version to emphasize the theme (Wang, 2021). This raises the question of why translators do not normalize the use of pronouns and other functional word categories in technical translation.

The motivations behind these decisions likely stem from the translators’ efforts to balance their efficiency with the clarity needed by readers. The English-text-driven explicitation of pronouns, termed ‘Source Language Shine Through’ in English-Chinese translation, reflects the syntactic structure of the original English content. This is because pronoun usage aligns well with lexical and syntactic norms in Chinese. Hence, to optimize their own efficiency and minimize their effort during translation, translators may opt to utilize this strategy, even if it results in a slight increase in the pronoun ratio, diverging from the ideal of normalization.

However, the efficiency and understanding of readers cannot be entirely overlooked, and professional translators need to find a middle ground. Translators make use of considerable effort into rephrasing sentences that diverge from direct lexical and syntactic correspondence. This is evident in Example 4, where they omitted the pronoun “we (我们)” in the translation. It is this dynamic equilibrium between the efficiency of translators and that of readers that dictates the translators’ approach to pronouns, transitions, and adpositions. While normalization is a goal, it may sometimes be set aside due to the demands placed on professional translators.

## Conclusion

The study explored translation universals using a corpus comprising articles sourced from the Communications of CCF, focusing on its primary audience of engineers and researchers in the field of computing. The results of the study suggest that more comprehensive corpus-based studies of technical English variables are needed to identify influential dimensions and develop advanced analytical tools. Furthermore, the concepts of entropy and the balanced effort hypothesis warrant deeper investigation, especially in the context of translators’ psychological activities. The findings of the study reveal valuable insights into the presence of translation universals and their driving factors, though the study’s scope is limited to professional computing translators. Thus, the conclusions drawn here may require validation in other domains or in the works of different professional translators. In sum, we argue that the empirical translation studies are ripe for rejuvenation. Moving beyond disciplinary silos, researchers should broaden their horizons by expanding the few established operationalizations of explicitness (e.g., alternation, cohesive devices, full vs. contracted forms), simplification (e.g., lexical density, average sentence length), and normalization (e.g., lexical bundles) that have been prevalent for over two decades; exploring new, nuanced linguistic indicators; and investigating other phenomena that might offer deeper insights into the nature of translation universals.

## Data Availability

The raw data supporting the conclusions of this article will be made available by the authors, without undue reservation.
